# Factors associated with hospitalizations for co-occurring HIV and opioid-related diagnoses: Evidence from the national inpatient sample, 2009–2017

**DOI:** 10.1016/j.pmedr.2023.102225

**Published:** 2023-05-09

**Authors:** Nima Khodakarami, Marvellous A. Akinlotan, Timothy Callaghan, Kristin M. Primm, Meera Vadali, Jane Bolin, Alva O. Ferdinand

**Affiliations:** a Pennsylvania State University, Department of Health Policy & Administration, United States; b Texas A&M University College of Nursing, United States; c Southwest Rural Health Research Center, Texas A&M University School of Public Health, United States; d Department of Health Law, Policy, and Management, Boston University School of Public Health, United States; e Department of Epidemiology, The University of Texas MD Anderson Cancer Center, United States; f Department of Health Policy & Management, Texas A&M School of Public Health, United States

**Keywords:** Opioid misuse, Hospitalization, HIV infections, Humans, Inpatient care, Rurality

## Abstract

There has been evidence of rising HIV incidence attributable to opioid misuse within some areas of the U.S. The purpose of our study was to explore national trends in co-occurring HIV and opioid-related hospitalizations and to identify their risk factors. We used the 2009–2017 National Inpatient Sample to indicate hospitalizations with co-occurring HIV and opioid misuse diagnoses. We estimated the frequency of such hospitalizations per year. We fitted a linear regression to the annual HIV-opioid co-occurrences with year as a predictor. The resulting regression did not reveal any significant temporal changes. We used multivariable logistic regression to determine the adjusted odds (AOR) of hospitalization for co-occurring HIV and opioid-related diagnoses. The odds of hospitalization were lower for rural residents (AOR = 0.28; CI = 0.24–0.32) than urban. Females (AOR = 0.95, CI = 0.89–0.99) had lower odds of hospitalization than males. Patients identifying as White (AOR = 1.23, CI = 1.00–1.50) and Black (AOR = 1.27, CI = 1.02–1.57) had higher odds of hospitalization than other races. When compared to co-occuring hospitalizations in the Midwest, the odds were higher in the Northeast. (AOR = 2.56, CI = 2.07–3.17) Future research should explore the extent to which similar findings occur in the context of mortality and targeted interventions should intesify for subpopulations at highest risk of co-occuring HIV and opioid misuse diagnoses.

## Highlights

• Rural residence is protective of experiencing a hospitalization for both HIV and opioid-related conditions.
• Residents of the Northeast were 2.5 times more likely to be hospitalized for co-occurring HIV and opioid when compared to residents of the Midwest.
• Patients which were identified as White, or Black were more likely than other races to experience hospitalizations for both HIV and opioid-related conditions.
• Patients with no insurance, Medicaid, or other public coverages were more likely than privately insured patients to be hospitalized for co-occurring HIV and opioid-related conditions.

## 1 Introduction

Opioid use and misuse in the United States (U.S.) has reached epidemic levels in the past 12 years (Health and Human Services, 2021, Shipton et al., 2018). Increases in opioid misuse have put users at risk for injection drug use (IDU) and related diseases like human immunodeficiency virus (HIV) (Degenhardt et al., 2019, Levitt et al., 2020). According to a recent report, from 2012 to 2016, unsafe injection practices resulted in about 2,300 annual HIV infections (Centers for Disease Control and Prevention 2018). Given that opioid misuse is projected to continue at a national level (Canady 2018), concerns are reinforced about the increased spread of HIV in conjunction with the opioid epidemic (Langabeer et al., 2020, McAuley et al., 2019).

Historical U.S. HIV surveillance data have shown that most HIV-positive injection-drug users were Black, older than 35 years of age, male, and urban residents (Lyss et al. 2018). However, in recent years, patterns of HIV infections have changed, as more HIV diagnoses attributed to IDU have occurred among those identifying as White, young persons, and residents of counties outside the large central metropolitan areas (Lyss et al., 2018, Van Handel et al., 2016, Ludwig et al., 2021, Mateu-Gelabert et al., 2015, Schranz et al., 2021). For example, a study of the HIV outbreak in rural Scott County-Indiana showed that the 181 injection drug users diagnosed with HIV were mostly young White persons, with almost half being women (Strathdee and Beyrer 2015). An analysis of HIV transmission attributable to IDU in West Virginia showed that diagnosed persons were mostly non-Hispanic White persons, male, and younger than 35 (Bradley et al. 2019). Another study of people diagnosed with HIV attributable to IDU in northeastern Massachusetts showed that affected persons were mostly non-Hispanic White, female, and aged 20 to 39 years. From 2014 to 2018, the rate of HIV diagnoses attributable to IDU among women and Whites increased by 7% and 26% respectively (Centers for Disease Control and Prevention 2019a). Despite this alarming evidence (Centers for Disease Control and Prevention 2020), the extent to which current findings are generalizable to the U.S. as a whole is unknown. Notably, the aforementioned studies were done in specific states and did not make use of nationally representative data. Given the rapidity with which HIV can spread when introduced into a community of IDU (Lyss et al. 2018), having information about the sub-populations at risk is needed.

Therefore, the purpose of this study is to explore how national trends in HIV and opioid-related hospitalizations have changed in light of the opioid epidemic and to identify the demographic and socioeconomic characteristics associated with persons hospitalized with co-occurring HIV and opioid diagnoses. Because over half of infected patients experience hospitalization (Randall et al. 2022), we used a dataset that is representative of U.S. hospitalizations. In so doing, this work will uncover the extent to which factors such as rurality, insurance coverage, demographic, and socioeconomic status play roles in the likelihood of diagnosis for both conditions during a hospital stay. Given the important role of hospitals in providing care for patients in the current opioid crisis (Song, 2017, Bassetti et al., 2002), this study offers evidence of hospitalization for HIV patients who misuse opioids, which is crucial for forecasting and developing targeted strategies (Laine et al., 2001, Bellino et al., 2019). Overall, this study will be of interest to clinicians, public health professionals, insurance executives, policy makers, and stakeholders who are interested in reducing harms associated with opioid use and misuse, such as HIV infection.

## 2 Material and methods

We conducted a retrospective cross-sectional study to explore predictors of hospitalizations for co-occurring HIV and opioid diagnoses. We used the Healthcare Cost and Utilization Project's (HCUP's) National Inpatient Sample (NIS) discharge records of patients 19 years old and above. The NIS is the largest publicly available all-payer inpatient database in the U.S. It contains data from over 3000 hospitals and includes around 7 million unweighted hospital stays each year. The large sample size of the NIS makes it ideal for developing national estimates of rare conditions, and special populations (Healthcare Cost and Utilization Project 2022a). The NIS is also an ideal dataset for analysis of hospitalization trends (Stulberg and Haut 2018) and is assumed to provide reliable estimates of conditions within the U.S. (Adejumo et al. 2017).

For this study, we used the NIS from 2009 through 2017. This period covers the time that recent waves of the U.S. opioid epidemic started (i.e., 2010 & 2013), and up to the year the opioid crisis was deemed to be a national crisis (i.e., 2017). The NIS contains clinical and nonclinical data elements on all hospital stays, including one primary diagnosis and up to twenty-four secondary diagnoses for each hospitalization record. Depending on the year, the coding of diagnoses is based on the ninth or tenth version of the International Classification of Diseases (ICD). Beginning October 1, 2015, ICD-10-CM replaced ICD-9-CM. We have included the ICD-9-CM and ICD-10-CM diagnosis codes for identifying HIV and opioid-related cases in this study in appendix 1.

### 2.1 Outcomes and exposure variables

We created three binary variables for each discharge record. We queried all-listed diagnoses within each discharge for existence of HIV and opioid-related conditions. Our primary outcome variable included both HIV and opioid-related conditions within a discharge. The variable was set to one if a discharge had both conditions, and zero otherwise. The other two binary variables were created where the discharge had just one of the two conditions (i.e., either HIV or opioid). The diagnoses codes we used for identification of opioid-related conditions are available in the HCUP case study by Moore and Barrett (2017).

Our primary exposure variable was rurality. This was based on recent findings that U.S. rural residents and communities have been affected disproportionately by opioid misuse as compared to their urban or metropolitan counterparts (Palombi et al. 2018). We indicated all non-metropolitan counties including micropolitan and noncore counties as rural (Office of Management and Budget, 2006, Health and Human Services Defining Rural Population, 2022). In selection of other exposures, we followed previous literature and included sex, age, race and ethnicity, insurance, income, and census regions (Van Draanen et al. 2020). The crude age for each record was available and we categorized it into four groups: 19–24, 25–44, 45–64, and 65 years and above. We used this grouping to follow the HCUP report that translated ICD-9-CM coding for opioid-related diagnosis to ICD-10-CM across age groups (Moore and Barrett 2017). For the patient’s race or ethnicity, we used four categories including White, Black, Hispanic, and Others, including Asian or Pacific Islander, Native American, and multiple races. We categorized insurance into five groups including Medicare, Medicaid, private insurance, uninsured, and other (government programs).

### 2.2 Statistical analysis

To statistically test for a significant linear trend, we fitted a linear regression to the annual HIV-opioid co-occurring hospitalizations with year as a predictor. In illustrating trends by selected risk factors, we estimated the adjusted count of HIV and opioid-related hospitalizations per 100,000 population by using the 2010 Census population (USCB 2010). Next, we evaluated the adjusted odds ratio (AOR) by running a multivariate logistic regression to assess the association between the above-listed outcome variables and various categorical exposures. A p-value of 0.05 was set as the cutoff for statistical significance. In our statistical model, we controlled for discharge year and severity of illness using the Charlson Comorbidity Index (CCI) (Charlson et al. 1987). To estimate the number of hospital discharges nationwide with HIV and opioid-related diagnosis, we weighted the data by using the weight variable (DISCWT) and stratum identifier (NIS_STRATUM) provided in the NIS. The stratum identifier is used to post-stratify hospitals for the calculation of universe and frame weights that includes the hospital's census division, ownership/control, location/teaching, and bed size (Healthcare Cost and Utilization Project, 2008). We used STATA 15 in conducting analyses. This study was determined to be exempt from oversight of research involving human subjects by the Texas A&M University Institutional Review Board.

## 3 Results

Of a total of 4,680,408 (weighted: 23,015,744) discharge records, we found 16,980 (weighted: 84,193) discharges with co-occurring HIV and opioid-related diagnoses (Table 1). Geographically, most discharges for co-occurring HIV and opioid misuse were for residents of large central metropolitan (non-rural) areas (66%), residents of the Northeast (57%), and those who lived in the lowest income zip Code quartile (55%). Also, a majority were male (62%), and between 45 and 64 years old (67%). Population characteristics for HIV and opioid-related diagnoses are presented in Table 1.

**Table 1:** Socio-demographic characteristics and other risk factors among patient discharge records classified by HIV and opioid-related diagnosis, 2009 to 2017 (counts are weighted).

	HIV-Opioid % (n = 84,193)	Opioid alone % (n = 5,862,058)	HIV alone % (n = 1,168,345)
***Location***			
Metro^1^	98% (n = 82,509)	87% (n = 5,099,990)	94% (n = 1,098,244)
Rural^2^	2% (n = 1,684)	13% (n = 762,068)	6% (n = 70,101)
***Sex***			
Male	62% (n = 52,200)	51% (n = 2,989,650)	68% (n = 806,158)
Female	38% (n = 31,993)	49% (n = 2,872,408)	32% (n = 362,187)
***Age Group***			
Age 19–24	1% (n = 842)	9% (n = 527,585)	3% (n = 35,050)
Age 25–44	29% (n = 24,416)	40% (n = 2,344,823)	35% (n = 420,604)
Age 45–64	67% (n = 56,409)	38% (n = 2,286,203)	55% (n = 642,590)
Age 65+	3% (n = 2,526)	13% (n = 762,068)	7% (n = 81,784)
***Race and Ethnicity***			
White	26% (n = 21,890)	67% (n = 3,927,579)	27% (n = 315,453)
Black	42% (n = 35,361)	15% (n = 879,309)	52% (n = 607,539)
Hispanic	19% (n = 15,997)	8% (n = 468,965)	12% (n = 140,201)
Others^3^	13% (n = 10,103)	10% (n = 586,206)	9% (n = 105,151)
***Insurance***			
Medicare	24% (n = 20,206)	29% (n = 1,699,997)	32% (n = 385,554)
Medicaid	61% (n = 51,358)	37% (n = 2,168,961)	41% (n = 455,655)
Private	7% (n = 5,894)	19% (n = 1,113,791)	15% (n186,935)
Uninsured	6% (n = 5,052)	11% (n = 644,826)	9% (n = 105,151)
Others^4^	2% (n = 1,684)	4% (n = 234,482)	3% (n = 35,050)
***Household Median Income***^5^			
First Quartile	55% (n = 46,306)	35% (n = 2,051,720)	50% (n = 584,173)
Second Quartile	20% (n = 16,839)	25% (n = 1,465,515)	23% (n = 268,719)
Third Quartile	16% (n = 13,471)	22% (n = 1,289,653)	17% (n = 198,619)
Fourth Quartile	9% (n = 7,577)	18% (n = 1,055,170)	11% (n = 128,518)
***Hospital census region***			
Northeast	57% (n = 47,990)	26% (n = 1,524,135)	28% (n = 303,770)
Midwest	9% (n = 7,577)	22% (n = 1,289,653)	12% (n = 140,201)
South	23% (n = 19,364)	32% (n = 1,875,859)	45% (n = 537,439)
West	11% (n = 9,261)	20% (n = 1,172,412)	15% (n = 186,935)
***Year***			
2009	13.9% (n = 11,721)	7.8% (n = 455,116)	12.9% (n = 150,953)
2010	14.3% (n = 12,080)	8.7% (n = 509,330)	13.9% (n = 162,042)
2011	10.9% (n = 9,161)	9.1% (n = 532,318)	11.7% (n = 136,131)
2012	9.7% (n = 8,200)	9.4% (n = 550,890)	10.5% (n = 123,095)
2013	9.6% (n = 8,070)	9.7% (n = 566,605)	10.2% (n = 119,130)
2014	9% (n = 7,570)	10.3% (n = 603,215)	9.9% (n = 115,935)
2015	9.9% (n = 8,310)	12.3% (n = 719,500)	9.9% (n = 115,400)
2016	11.5% (n = 9,720)	16.3% (n = 953,229)	10.3% (n = 120,760)
2017	11.1% (n = 9,360)	16.6% (n = 971,855)	10.7% (n = 124,900)

1 Large central, large fringe, medium, and small metropolitan counties.

2 Micropolitan and Noncore counties.

3 Asian/ Pacific Islanders, Native American, multi-race, and others.

4 Other government, Indian Health Service, other race.

5 Annual income stratified by residence zip-code; the value ranges vary by year; the quartiles indicate the poorest to wealthiest populations.

**Fig. 1:**
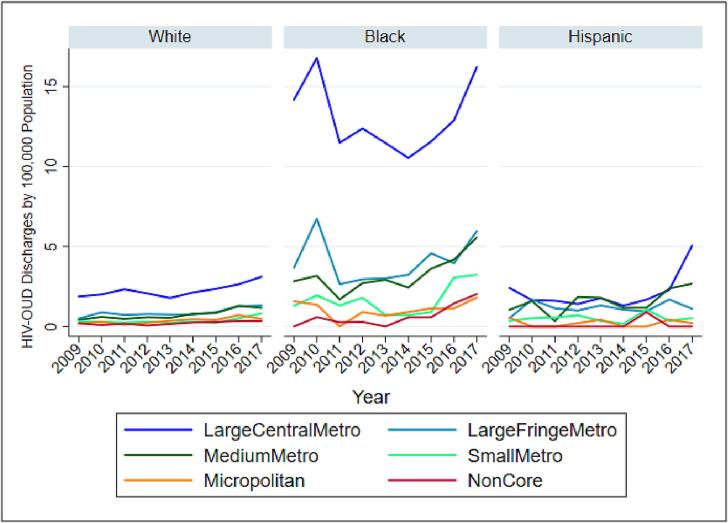
HIV-Opioid related hospitalizations by race.

**Fig. 2:**
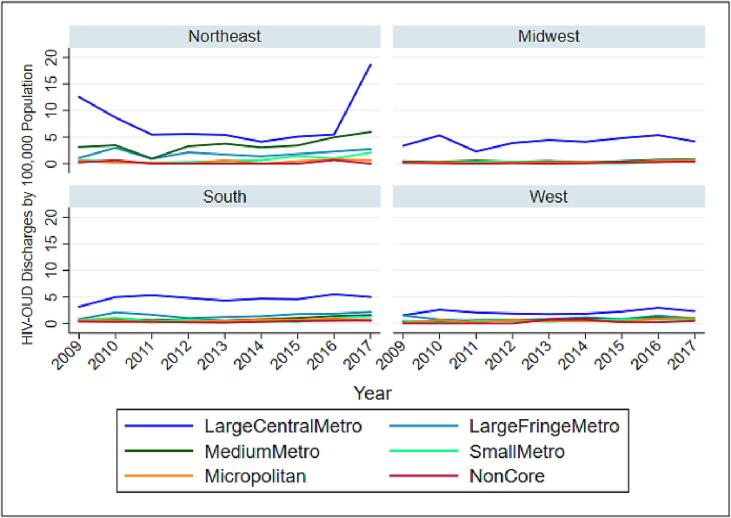
HIV-Opioid related hospitalizations by region.

**Fig. 3:**
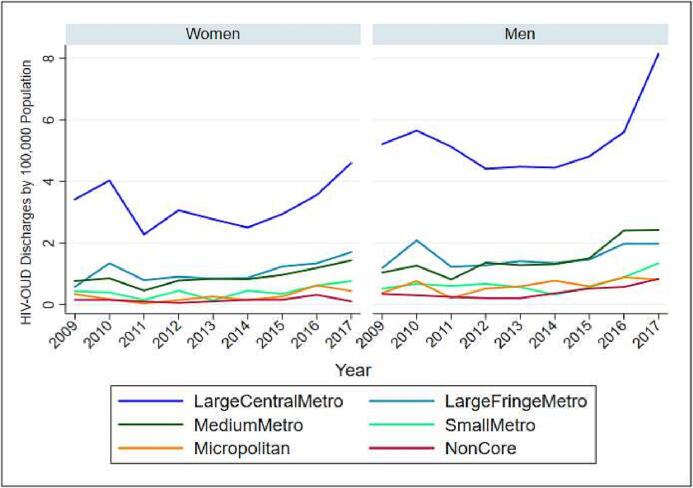
HIV-Opioid related hospitalizations by gender.

The linear trend did not reveal any significant temporal changes. However, this trend could be confounded with changes implemented in the 2012 HCUP-NIS redesign (Chen and Yoon 2016), and the transition to ICD-10-CM in October 2015 (Healthcare Cost and Utilization Project 2022b). We graphed population-adjusted counts of co-occurring HIV-opioid hospitalizations stratified by sex, race, and census regions across time in Fig. 1, Fig. 2, Fig. 3. An additional illustration of the weighted count of HIV-opioid cases by median household income quartile for patient zip codes is presented in the appendix 2.

### 3.1 The relationship between hospitalizations and risk factors.

We measured the AOR to estimate a relationship between our primary outcome of a discharge with co-occurrences of HIV-opioid and a primary exposure of rurality, as well as other risk factors including sex, age, race, insurance coverage, income, and hospital location by census regions. We also estimated the AOR for opioid discharges, as well as HIV discharges in the context of the above-listed risk factors. We found that the odds of diagnoses with co-occurring HIV and opioid conditions were lower among rural residents, as compared to their urban counterparts (AOR = 0.28; CI = 0.24–0.32). Similar results were found for just opioids or HIV hospitalizations. For other exposures, we found the following results. Females had slightly lower odds of diagnoses with HIV and opioid co-occurrences as compared to males (AOR = 0.95; CI = 0.89–0.99). When compared to 19–24 years old, we found higher odds of diagnoses with co-occurring HIV and opioid conditions among the older groups of 25 to 44 (AOR = 2.75; CI = 2.25–3.37) and 45 to 64 year olds (AOR = 2.33; CI = 1.889–2.88), but lower odds for those aged 65 years and above (AOR = 0.22; CI = 0.17–0.28). The results were different for hospitalizations with just opioid or HIV diagnoses individually. For opioid misuse hospitalizations, the odds of diagnoses were higher for those aged 25 to 44 (AOR = 1.23; CI = 1.19–1.26), and lower for those 65 years and above (AOR = 0.20; CI = 0.19–0.21) as compared to 19–24 year olds. For HIV discharges, the odds of diagnoses were lower for all age groups when compared to 19–24 year olds. Compared to other races, the odds of diagnoses with HIV-opioid co-occurrences were significantly higher for Whites (AOR = 1.23; CI = 1.00–1.50) and Blacks (AOR = 1.27; CI = 1.02–1.57). In comparison, the odds of a hospitalization for just opioid misuse were higher for Whites (AOR = 1.53; CI = 1.45–1.62) and lower for Blacks (AOR = 0.89; CI = 0.82–0.98) and Hispanics (AOR = 0.63; CI = 0.59–0.67). On the contrary, the odds of diagnosis for just HIV were lower for Whites (AOR = 0.74; CI = 0.66–0.83), higher for Blacks (AOR = 2.46; CI = 2.20–2.76) and higher for Hispanics (AOR = 1.19; CI = 1.07–1.33). Compared to the privately insured, those who were uninsured or covered by other types of insurance had higher odds of hospitalization for co-occurring HIV and opioid diagnoses. The odds of hospitalizaitons for co-occurring HIV and opioid diagnoses, as well as hospitalizations for either opioid misuse or HIV were higher for patients living within poorer areas (Table 2). When compared to hospitalizaitons occuring in the Midwest, the odds of co-occurring HIV and opioid diagnoses were significantly higher in the Northeast (AOR = 2.56; CI = 2.07–3.17) and the West (AOR = 1.17; CI = 0.99–1.37). In the context of hospitalizations for just opioid misuse, the odds were higher in the Northeast (AOR = 1.25; CI = 1.59–1.35) and the West (AOR = 1.09; CI = 1.02–1.16), and lower in the South (AOR = 0.77; CI = 0.72–0.82). Moreover, the odds of hospitalization with just HIV diagnoses were higher in the South (AOR = 2.25; CI = 2.08–2.42) and the West (AOR = 1.84; CI = 1.65–2.05) when compared to the Midwest (Table 2).

**Table 2:** Adjusted odds ratio (AOR) for HIV and Opioid-related diagnoses, 2009–2017.

	HIV-Opioid	Opioid	HIV
Patient location (*Ref. metro area*)			
Rural	0.28***	0.69***	0.49***
	(0.02)	(0.01)	(0.01)
Sex (*Ref. male*)			
Female	0.95**	0.65***	0.37***
	(0.03)	(0.00)	(0.01)
Age (*Ref. age 19*–*24*)			
Age 25–44	2.75***	1.23***	0.95*
	(0.28)	(0.02)	(0.03)
Age 45–64	2.33***	0.98	0.21***
	(0.25)	(0.02)	(0.00)
Age 65+	0.22***	0.20***	0.01***
	(0.03)	(0.00)	(0.00)
Race (*Ref. other races*)			
White	1.23**	1.53***	0.74***
	(0.11)	(0.04)	(0.04)
Black	1.27**	0.89**	2.46***
	(0.12)	(0.04)	(0.14)
Hispanic	1.21	0.63***	1.19***
	(0.13)	(0.02)	(0.06)
Insurance (*Ref. private coverage*)			
Medicare	1.93***	2.83***	1.29***
	(0.15)	(0.03)	(0.02)
Medicaid	3.86***	3.28***	1.62***
	(0.35)	(0.06)	(0.03)
Uninsured	3.02***	3.33***	2.30***
	(0.35)	(0.09)	(0.07)
Other Pay	1.78***	1.97***	1.52***
	(0.20)	(0.05)	(0.06)
Household Median Income (*Ref. income Fourth Quartile*)			
First Quartile	1.82***	1.31***	1.48***
	(0.12)	(0.02)	(0.06)
Second Quartile	1.37***	1.09***	1.27***
	(0.09)	(0.02)	(0.05)
Third Quartile	1.20***	1.04***	1.08*
	(0.06)	(0.01)	(0.04)
Census region (*Ref. Midwest)*			
Northeast	2.56***	1.25***	1.02
	(0.27)	(0.05)	(0.05)
South	0.95	0.77***	2.25***
	(0.09)	(0.03)	(0.08)
West	1.17*	1.09***	1.84***
	(0.09)	(0.03)	(0.10)

Standard errors are in the parentheses.

Odd ratios are presented at * p < 0.10, ** p < 0.05, *** p < 0.01 significance levels.

## 4 Discussion

In this study, we set out to explore the extent to which trends in HIV and opioid-related hospitalizations have changed over time and the role that sociodemographic factors played in predicting hospitalizations for co-occurring HIV and opioid-related diagnoses between 2009 and 2017. We note a few main findings. The first is that we did not discern a significant change in hospitalization trends for co-occurring HIV and opioid during the study period. Our finding aligns with the most recent HIV surveillance report that determined that from 2014 to 2018, overall HIV diagnoses remained stable among persons who injected drugs. Notably, the report indicated increases of IDU-related HIV diagnoses among Whites and women (Centers for Disease Control and Prevention 2019a).

Second, while rural communities have been most discussed in terms of HIV outbreaks as a result of shared drug paraphernalia (Artherhults, 2021, Sun, 2016, Deppen, 2016, Subbaraman, 2018), our findings suggest that those residing in rural areas were less likely to be hospitalized for co-occurring HIV and opioid-related diagnoses. Importantly, our findings may be tempered by the fact that many rural communities have experienced hospital closures, and therefore the opportunity for hospitalization may be diminished as a result (Government Accountability Office 2020). Thus, it is conceivable that though rural residents are less likely to experience hospitalization for co-occurring HIV and opioid-related diagnoses, they are more likely to experience mortality from these conditions. Notably, in this study, we did not examine HIV and opioid-related mortality. However, it has been noted in previous descriptive work that rural residents die less frequently in this context than their urban counterparts (Callaghan et al. 2021). Overall, our results align with previous findings that showed heroin overdose hospitalizations were lower in rural areas compared to urban (Mosher et al. 2017).

Third, though Black patients have the highest odds of experiencing an HIV-related hospitalization, White patients have comparable odds of being hospitalized for both HIV and opioid-related diagnoses as Black patients. This finding seems to align with that of other researchers who have noted that opioid prescriptions have disproportionately been filled by White patients, and this has in many cases led to the concern that they may subsequently turn to heroin (Hansen and Netherland 2016). According to the National Institute on Drug Abuse, the risk of being exposed to HIV increases with heroin use because of contact with infected blood and other bodily fluids through shared syringes and injection paraphernalia (National Institute on Drug Abuse 2021a). Thus, our finding that White patients have high likelihood of acquiring and obtaining inpatient care for co-occurring HIV and opioid misuse conditions suggests that there is an urgent need for harm reduction programs and increased availability of addiction treatment for this group. Importantly, patients identifying as Black were also at increased odds of undergoing such a hospitalization relative to other races, indicating that addiction treatment and harm reduction programs should also be made more widely available for this minority group. Our findings suggest that a shift in U.S. drug policy that is more inclusive and not primarily focused on incarceration for nonviolent illicit drug offenses, particularly among individuals identifying as Black, is critical for combatting the opioid epidemic and mitigating spikes in the prevalence of HIV that occur because of it. Hospitalization for these co-occurring conditions supports the idea that harm reduction may not only prevent opioid-related hospitalizations but reduce HIV infection and subsequent hospitalizations as well.

Fourth, while it is generally known that the South bears the brunt of most health maladies in the U.S. (Maddock 2018), and while the odds of an HIV-related hospitalization are highest in the South, residents of the Northeast were much more likely to experience hospitalization for co-occurring HIV and opioid-related diagnoses relative to residents of the Midwest. This finding is perhaps surprising given the fact that the 16 states that make up the U.S. South are the epicenter of the HIV epidemic in the U.S. (Centers for Disease Control and Prevention 2019b), with the South accounting for more than half of all HIV diagnoses in the U.S. in 2019 (Kaiser Family Foundation 2021). Nevertheless, it has been noted that most states in New England have been seeing high opioid-related death rates and high rates of suspected opioid overdose (National Institute on Drug Abuse 2021b). Thus, our finding that co-occurring HIV and opioid-related hospitalizations are mostly likely to occur in the Northeast should be noted. Importantly, the federal government has already specifically allocated funds to treat heroin abuse in the Northeast (Shear 2015). Our findings suggest that efforts and resources to combat HIV that is acquired via injection drug use should be carefully considered in this region as well.

We note a few limitations of our work. This work was largely reliant on diagnosis codes, which are inherently subject to human error. Moreover, the NIS is discharge-specific and not patient-specific, making it impossible to identify instances in which one patient had more than one admission. Further, because we relied on the NIS, only in-hospital outcomes could be examined. It is widely known that many opioid-related outcomes occur outside of the hospital setting (Ogeil et al., 2018, Siegler et al., 2014). Nevertheless, diagnosis codes have been routinely used in health services research to examine trends and associations. Another limitation of the NIS is that it does not contain indicators for conditions that were present on admission thereby making it impossible to determine what diagnoses were acquired during hospitalization (Mori et al. 2019). Therefore, it is possible that the discharges we analyzed contained some hospital-acquired diagnoses. However, the Centers for Disease Control and Prevention (CDC) has stated that HIV transmission is extremely rare in healthcare settings (Centers for Disease Control and Prevention 2021). Given that the opioid epidemic is still ongoing, another limitation in studying recent trends is that our data availability stopped in 2017. Future work should explore the extent to which trends have shifted in more recent years.

Despite these limitations, this study provides critical information on the sub-populations of the U.S. that are more likely to require hospitalization for co-occurring HIV and opioid use disorder diagnoses. Importantly, our findings indicate that targeted interventions are needed to reduce the incidence of hospitalizations among people living with both HIV and opioid use disorder, particularly among urban residents, residents of the Northeast, Whites, and minority populations, as well as those residing in low-income communities. While reports of outbreaks of HIV among IDUs have largely focused on rural areas, our findings indicate that rural residents are significantly less likely to experience hospitalization for these conditions. Future research should explore the extent to which rural residence is also associated with a lower risk of mortality tied to co-occurring HIV infection and opioid use disorder.

## Funding sources

This study was supported by the Federal Office of Rural Health Policy (FORHP), Health Resources and Services Administration (HRSA), U.S. Department of Health and Human Services (HHS) under cooperative agreement #U1CRH30040. The information, conclusions, and opinions expressed in this brief are those of the authors and no endorsement by FORHP, HRSA, or HHS is intended or should be inferred.

## Contributors

All authors have been personally and actively involved in substantive work leading to the report and will hold themselves jointly and individually responsible for the manuscript’s content.

## Declaration of Competing Interest

The authors declare that they have no known competing financial interests or personal relationships that could have appeared to influence the work reported in this paper.

## Data Availability

The authors do not have permission to share the data.
