# Supplementary material for: Factors associated with hospitalizations for co-occurring HIV and opioid-related diagnoses: Evidence from the national inpatient sample, 2009–2017
Source: Prev Med Rep. 2023 May 9;34:102225. doi: 10.1016/j.pmedr.2023.102225 (PMC10199244; doi:10.1016/j.pmedr.2023.102225)
Supplement: Supplementary data 1 [file mmc1.docx]

**Appendix 1:** ICD-9-CM and ICD-10-CM Diagnosis Codes Used in the Study

|  | **ICD-9** | **ICD-10** |
| --- | --- | --- |
| **HIV** | 042 | B20-B24 |
| **Opioid** |  |  |
| Opioid Abuse | 30550-30552 | F1110; F11120-F11122; F11129; F1114; F11150-F11151; F11159; F11181-F11182; F11188; F1119 |
| Adverse Effects of Opioids | E9350-E9352; E9401 | T400X5A; T400X5D; T400X5S; T402X5A; T402X5D; T402X5S; T403X5A; T403X5D; T403X5S; T404X5A; T404X5D; T404X5S; T40605A; T40605D; T40605S; T40695A; T40695D; T40695S |
| Opioid Dependence and Unspecified Use | 30400-30402; 30470-30472 | F1120; F11220; F11221; F11222; F11229; F1123; F1124; F11250; F11251; F11259; F11281; F11282; F11288; F1129; F1190; F11920; F11921; F11922; F11929; F1193; F1194; F11950; F11951; F11959; F11981; F11982; F11988; F1199 |
| Opioid Poisoning | 96500-96502; 96509; 9701; E8500-E8502 | T400X1A; T400X1D; T400X1S; T400X4A; T400X4D; T400X4S; T401X1A; T401X1D; T401X1S; T401X4A; T401X4S; T401X4D; T402X1A; T402X1S; T402X1D; T402X4A; T402X4D; T402X4S; T403X1A; T403X1D; T403X1S; T403X4A; T403X4D; T403X4S; T404X1A; T404X1S; T404X1D; T404X4A; T404X4S; T404X4D;T40601A; T40601D; T40601S; T40604A; T40604D; T40604S; T40691A; T40691D; T40691S; T40694A; T40694D; T40694S |

*Selection of the HIV-opioid related cases was based on the above table. If a patient was admitted to a healthcare facility with HIV listed as their primary diagnosis and opioid condition listed as an additional diagnosis, they were considered to have co-occurring HIV-opioid conditions. If a patient was admitted to a healthcare facility with opioid listed as their primary diagnosis and HIV condition listed as an additional diagnosis, they were considered to have co-occurring HIV-opioid conditions.*

**Appendix 2:** Weighted Count of HIV-Opioid by Median Household Income Quartile For Patient Zip Code


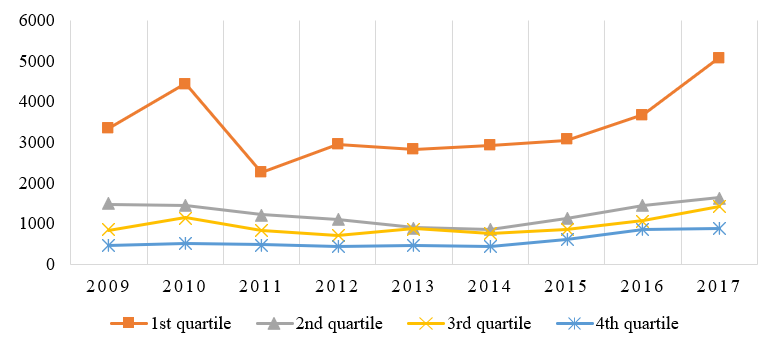


Weighted Count of HIV-Opioid

*Note:* *The 1st quartile captured the poorest hospitalized patients and the 4th quartile the wealthiest patients. For example, in 2017 the 1^st^ quartile captured individual whose income was between $1-43,999, and the 2^nd^ quartile captured individual whose income was between $ 44,000 - 55,999, and the 3^rd^ quartile captured individual whose income was between $ 56,000 - 73,999, and the 4^th^ quartile captured individual whose income was above $ 74,000.*
